# Molecular characterization of two novel reoviruses isolated from Muscovy ducklings in Guangdong, China

**DOI:** 10.1186/s12917-019-1877-x

**Published:** 2019-05-10

**Authors:** Xue-Lian Zhang, Jian-Wei Shao, Xiao-Wen Li, Min-Min Mei, Jin-Yue Guo, Wen-Feng Li, Wen-Jing Huang, Shi-Hong Chi, Sheng Yuan, Zhi-Li Li, Shu-Jian Huang

**Affiliations:** 1 Key Laboratory for Preventive Research of Emerging Animal Diseases, Foshan University, Foshan, 528231 Guangdong China; 2 College of Life Science and Engineering, Foshan University, Foshan, 528231 Guangdong China; 3 Department of Medical Microbiology and Parasitology, Zhejiang University School of Medicine, Zhejiang, China

**Keywords:** Novel Muscovy duck reovirus, Pairwise sequence comparison, Phylogenetic analysis, Re-assortment analyses

## Abstract

**Background:**

Novel Muscovy duck reovirus (N-MDRV), emerged in southeast China in 2002, which can infect a wide range of waterfowl and induces clinical signs and cytopathic effects that are distinct from those of classical MDRV, and continues to cause high morbidity and 5–50% mortality in ducklings. The present study aimed to investigate the characteristics of two novel reoviruses isolated from Muscovy ducklings in Guangdong, China.

**Results:**

Two novel MDRV strains, designated as MDRV-SH12 and MDRV-DH13, were isolated from two diseased Muscovy ducklings in Guangdong province, China in June 2012 and September 2013, respectively. Sequencing of the complete genomes of these two viruses showed that they consisted of 23,418 bp and were divided into 10 segments, ranging from 1191 bp (S4) to 3959 bp (L1) in length, and all segments contained conserved sequences in the 5′ non-coding region (GCUUUU) and 3′ non-coding region (UCAUC). Pairwise sequence comparisons demonstrated that MDRV-SH12 and MDRV-DH13 showed the highest similarity with novel MDRVs. Phylogenetic analyses of the nucleotide sequences of all 10 segments revealed that MDRV-SH12 and MDRV-DH13 were clustered together with other novel waterfowl-origin reoviruses and were distinct from classical waterfowl-origin and chicken-origin reoviruses. The analyses also showed possible genetic re-assortment events in segment M2 between waterfowl-origin and chicken-origin reoviruses and the segments encoding λA, μA, μNS, σA, and σNS between classical and novel waterfowl-origin reoviruses. Potential recombination events detection in segment S2 suggests that MDRV-SH12 and MDRV-DH13 may be recombinants of classical and novel WRVs.

**Conclusions:**

The results presented in this study, the full genomic data for two novel MDRV strains, will improve our understanding of the evolutionary relationships among the waterfowl-origin reoviruses circulating in China, and may aid in the development of more effective vaccines against various waterfowl-origin reoviruses.

**Electronic supplementary material:**

The online version of this article (10.1186/s12917-019-1877-x) contains supplementary material, which is available to authorized users.

## Background

Avian reoviruses (ARVs) belong to the genus *Orthoreovirus* in the family *Reoviridae*, which includes mammalian reovirus (MRV), Nelson Bay reovirus (NBV), Baboon reovirus (BRV), and Reptilian reovirus (RRV) [1]. ARV is a double-stranded RNA (dsRNA) virus, and the genome consists of 10 segments packaged into a non-enveloped icosahedral double-capsid shell, with a diameter of 70–80 nm [1, 2]. The genome of ARV is comprised of three large (L1, L2, and L3), three medium (M1, M2, and M3), and four small (S1, S2, S3, and S4) segments, which encode proteins of the λ, μ, and σ classes, respectively [3–6]. The first seven bases (5′-GCUUUUU-3′) of the 5′ non-coding regions (NCRs) and the last five bases (5′-UCAUC-3′) of the 3′ NCR of each ARV genome segment are highly conserved across all ARV strains [7].

ARVs are important etiological agents; they can cause large economic losses in the poultry industry, as they infect a variety of domestic poultry and wild avian species, including chickens [8], turkeys [9], Muscovy ducks [10], Pekin ducks [11], geese [12], wild mallard ducks [13], pigeons [14], psittacine birds [15], and other wild birds. The signs of ARV infection in waterfowl include general weakness, diarrhea, serofibrinous pericarditis, and a swollen liver and spleen with small white necrotic foci [10, 16, 17]. Waterfowl-origin reovirus (WRV) was first identified as a pathogen in South Africa in 1950 [18]. It was subsequently isolated from Muscovy ducks in France in 1972 and was designated as classical Muscovy duck reovirus (MDRV) [10]. Classical MDRV first emerged in China in 1997. It mainly infects Muscovy ducklings at 10 days of age, and the infection persists until the ducklings are 6 weeks old. The mortality rate ranges from 10 to 30% [19]. However, many researchers have reported that classical MDRV isolates are non-pathogenic in shelduck ducklings, Pekin ducklings, and other duckling varieties [19, 20].

In 2002, a new infectious disease emerged in Muscovy ducklings in southeast China, which is the major Muscovy duck production area in China [20]. Unlike classical MDRV infection, this disease is mainly characterized by severe hemorrhagic, necrotic lesions in the liver and spleen, with a mortality rate ranging from 5 to 50%. The virus can infect a variety of duck species, such as Pekin, Muscovy, and domesticated wild duck. Goslings have also been infected in some parts of China [21–23]. Because of the different clinical signs and cytopathic effects compared to chicken-origin ARVs and classical MDRVs, the causative agent of this disease was named as novel Muscovy duck reovirus (N-MDRV). Phylogenetic analyses based on the amino acid sequences encoded by the S2 and S3 segments also demonstrated that N-MDRVs are significantly different from chicken-origin ARVs and classical MDRVs [24].

In this study, two novel field strains of duck reovirus, named MDRV-SH12 and MDRV-DH13, were isolated from two diseased Muscovy ducklings in Guangdong province, China in June 2012 and September 2013, respectively. To better understand the molecular characteristics of the reoviruses circulating in waterfowl populations, the whole genomes of these two viruses were cloned, sequenced, and analyzed. These complete genomic data may be helpful for understanding the evolutionary relationships among the WRVs and other orthoreoviruses circulating in China.

## Results

### Virus isolation

Two viruses, designated as MDRV-SH12 and MDRV-DH13, were isolated from clarified liver suspensions generated by three passages in the allantoic cavities of 10-day-old Muscovy duck embryos. Inoculation of duck embryos with the clarified liver suspensions resulted in 100% mortality within 48–120 h after inoculation, and severe subcutaneous hemorrhage was observed in the dead embryos. Based on the similarity of yellow necrotic foci in liver and spleen caused by other novel MDRVs, MDRV-SH12 and MDRV-DH13 were considered as two novel MDRV isolates.

### Complete genome sequences of MDRV-SH12 and MDRV-DH13

The complete sequences of the MDRV-SH12 and MDRV-DH13 genomes determined in this study were deposited into GenBank under accession numbers MH510245–MH510254 and MH510255–MH510264, respectively. The genomes of the two viruses were organized similarly to the genomes of the WRVs among *Avian orthoreovirus* species (Table 1). The complete genomes of MDRV-SH12 and MDRV-DH13 were 23,418 bp in length, and divided into the following 10 segments: L1 (3959 bp), L2 (3830 bp), L3 (3907 bp), M1 (2283 bp), M2 (2158 bp), M3 (1996 bp), S1 (1568 bp), S2 (1324 bp), S3 (1202 bp), and S4 (1191 bp). All 10 segments exhibited approximately 50% G + C content. To determine the coding regions of these two viruses, each segment sequence was used as a query in a BLASTp search of the non-redundant protein database. The proteins encoded by these two viruses included at least eight structural proteins (λA, λB, λC, μA, μB, σA, σB, and σC) and four nonstructural proteins (μNS, p10, p18, and σNS). These proteins were 97–1293 aa in length. ORF prediction and homology searches showed that 9 of the 10 genome segments encoded a single ORF. The exception of the S1 segments, which were polycistronic and each encoded three partially overlapping ORFs: p10 (20–313), p18 (273–761), and σC (571–1536).

**Table 1:** General genome features of two novel Muscovy duck reovirus strains SH12/DH13 isolated in Guangdong province of China

Genome segment	Size (bp)		Lengh (bp) of the 5’end-ORF-3′		Sequence at the termini 5′ end/3’end		Contig location	Protein size (aa)	Encoded protein
	SH12	DH13	SH12	DH13	SH12	DH13			
L1	3959	3959	21–3882-56	21–3882-56	GCUUUUU/UCAUC	GCUUUUU/UCAUC	22~3903	1293	λA (core shell)
L2	3830	3830	14–3780-36	14–3780-36	GCUUUUU/UCAUC	GCUUUUU/UCAUC	15~3794	1259	λB (core RdRp)
L3	3907	3907	12–3858-37	12–3858-37	GCUUUUU/UCAUC	GCUUUUU/UCAUC	13~3870	1285	λC (core turret)
M1	2283	2283	12–2199-72	12–2199-72	GCUUUUU/UCAUC	GCUUUUU/UCAUC	13~2211	732	μA (core NTPase)
M2	2158	2158	29–2028-101	29–2028-101	GCUUUUU/UCAUC	GCUUUUU/UCAUC	30~2057	675	μB (outer shell)
M3	1996	1996	24–1908-64	24–1908-64	GCUUUUU/UCAUC	GCUUUUU/UCAUC	25~1932	635	μNS (NS factory)
S1	1568	1568	19–1517-32	19–1517-32	GCUUUUU/UCAUC	GCUUUUU/UCAUC	20~313	97	p10 (NS -FAST)
							273~761	162	p18 (NS other)
							571~1536	321	σC (outer fibe)
S2	1324	1324	15–1251-58	15–1251-58	GCUUUUU/UCAUC	GCUUUUU/UCAUC	16~1266	416	σA (core clamp)
S3	1202	1202	30–1104-68	30–1104-68	GCUUUUU/UCAUC	GCUUUUU/UCAUC	31~1134	367	σB (outer clamp)
S4	1191	1191	23–1104-64	23–1104-64	GCUUUUU/UCAUC	GCUUUUU/UCAUC	24~1127	367	σNS (NS RNAb)

Comparison of the nucleotide sequences of these two viruses with other ARVs, for which all 10 full-length segments were available in GenBank, revealed that all 10 segments of these two viruses had different degrees of similarities with other ARVs. The viruses with the highest similarities (92–99%) to MDRV-SH12 and MDRV-DH13 were novel WRVs (ZJ00M, TH11, 091, NP03, HN5d, SD-12, J18, and 03G) isolated in China.

### NCRs of the MDRV-SH12 and MDRV-DH13 genome segments

Nucleotide sequence analysis of the 5′ and 3′ NCRs at the genome segment termini revealed that all 10 genome segments of these two viruses contained conserved nucleotides common to other ARVs (Table 1). The length of the NCRs was 13–30 bp at the 5′ ends, and 32–101 bp at the 3′ ends. All segments shared a GCUUUU motif at the 5′ NCR and a UCAUC motif at the 3′ NCR. These were highly conserved across all novel WRVs, classical WRVs, and other chicken-origin ARVs with available full-length segments (Table 1).

### Pairwise sequence comparisons

Based on the nt (97.1–99.8%) and aa (97.6–99.8%) sequence identity values, MDRV-SH12 and MDRV-DH13 were most similar to each other, and these two viruses were more closely related to WRVs than to chicken-origin ARVs (Table 2). Nucleotide sequence comparisons of the L-class genome segments, encoding λB and λC, revealed moderate to high sequence identities (87.5–99.5%) between these two viruses and other novel WRV isolates (ZJ00M, TH11, 091, NP03, HN5d, SD-12, J18, and 03G), and greater variation (70.1–76.9% identity) between these two viruses and chicken-origin ARVs. Comparison of the λA protein-coding gene L1 segment showed that these two viruses have higher sequence identities (85.4–98.8%) with WRVs than with chicken-origin ARVs (77.2–77.8%). However, comparison of MDRV-SH12 and MDRV-DH13 with novel WRVs showed higher sequence identities (93.7–98.8%) between these two viruses and ZJ00M, TH11, 091, HN5d, SD-12, and J18, than with two other novel waterfowl-origin reoviruses NP03 and 03G (86.3–86.8%). The amino acid sequence comparisons showed that these two viruses shared high sequence identities (91.0–99.8%) with waterfowl-origin and chicken-origin reoviruses, with the exception of the λC proteins, as these proteins shared lower identities (79.0–80.0%) with chicken-origin reoviruses.

**Table 2:** Sequence identities (%) of nt and aa between SH12/DH13 and other novel WRVs, classical WRVs, and chicken ARVs

Gene/Protein	SH12 vs novel WRVs		SH12 vs classical WRVs		SH12 vs chicken ARVs		SH12 vs DH13		DH13 vs novel WRVs		DH13 vs classical WRVs		DH13 vs chicken ARVs	
	nt	aa	nt	aa	nt	aa	nt	aa	nt	aa	nt	aa	nt	aa
λA	86.3–98.8	97.8–99.8	85.6–86.7	97.0–97.4	77.2–77.8	94.5–95.0	99.6	99.8	86.3–98.8	97.8–99.9	85.4–86.6	97.1–97.4	77.2–77.7	94.6–95.0
λB	87.5–98.2	97.2–99.5	87.3–88.3	96.9–97.9	75.6–76.9	91.0–91.2	97.7	99.0	87.1–98.2	97.0–99.4	87.5–88.3	96.7–97.8	75.4–77.0	90.7–91.0
λC	96.4–99.1	98.4–99.5	79.0–80.7	92.0–93.3	70.1–70.4	79.0–80.0	99.2	99.1	96.3–99.1	98.2–99.5	79.0–80.6	91.8–93.3	70.3–70.5	79.0–80.0
μA	95.9–98.5	97.1–99.5	79.7–95.1	90.8–97.0	73.3–73.6	85.9–87.0	98.0	98.9	95.6–98.2	96.9–99.0	79.8–94.9	91.0–96.7	73.5–73.7	86.2–87.0
μB	88.1–98.5	96.1–99.4	63.8–67.9	76.3–76.7	76.7–77.5	89.6–90.0	99.1	99.1	88.0–98.4	95.9–99.1	64.0–68.0	76.3–76.7	76.6–77.1	89.3–90.0
μNS	94.5–99.0	96.1–98.9	79.2–86.0	90.1–93.9	71.5–71.7	79.7–80.2	98.1	97.6	94.1–98.6	95.6–98.3	79.1–86.0	89.4–93.5	71.3–71.5	79.5–80.0
p10	95.6–99.0	99.0–100	38.1–39.5	9.6–10.8	53.5–54.7	33.0–34.0	98.6	100	95.6–99.0	99.0–100	37.4–38.8	9.6–10.8	37.9–54.4	33.0–34.0
p17/p18	98.3–99.5	93.9–98.3	–	–	10.9–11.6	14.9–17.1	99.4	97.8	98.2–99.5	93.9–98.3	–	–	10.9–11.6	15.5–16.6
σC	91.2–99.0	95.0–98.4	51.7–53.0	42.0–42.4	40.8–43.0	27.4–28.1	98.4	98.4	90.5–98.1	94.4–97.5	51.7–53.0	40.9–41.3	41.0–43.3	28.1–28.7
σA	88.2–99.4	97.6–100	87.2–95.4	96.4–99.0	76.8–77.5	90.4–91.8	99.1	99.5	87.3–99.1	97.1–99.5	86.6–94.9	95.9–98.6	76.8–77.5	89.9–91.3
σB	94.6–99.6	94.3–100	65.6–67.1	68.7–69.2	64.9–66.2	68.4–69.5	99.6	99.2	94.2–99.3	93.5–99.2	65.3–66.7	68.1–68.7	64.5–65.8	67.8–68.9
σNS	93.0–98.6	96.7–99.2	84.2–91.0	95.6–97.0	78.2–78.3	90.2–90.5	99.8	99.5	92.4–98.4	96.2–98.6	84.1–90.9	95.1–96.5	78.0–78.1	89.6–89.9

novel WRVs: waterfowl-origin reovirus isolates ZJ00M, TH11, 091, NP03, HN5d, SD-12, J18 and 03G

classical WRVs: waterfowl-origin reovirus isolates D20/99, 815–12, ZJ2000M, D1546 and D2044

chicken ARVs: chicken-origin reovirus isolates 138, C98, S1133 and T-98

*nt* nucleotide sequence

*aa* amino acid sequence

Comparison of the M-class genome segments (M1, M2, and M3) of MDRV-SH12 and MDRV-DH13 with other ARVs showed that these two viruses shared the highest sequence identities with novel WRVs (M1: nt, 95.6–98.5%; aa, 96.9–99.5%; M2: nt, 88.0–98.5%; aa, 95.9–99.4%; and M3: nt, 94.1–99.0%; aa, 95.6–98.9%). Unexpectedly, the M1 and M3 genome segments of these two viruses shared higher sequence identities with classical WRVs (M1: nt, 79.7–95.1%; aa, 90.8–97.0% and M3: nt, 79.1–86.0%; aa, 89.4–93.9%) than with chicken-origin ARVs (M1: nt, 73.3–73.7%; aa, 85.9–87.0%; M3: nt, 71.3–71.7%; aa, 79.5–80.2%), while the M2 genome segments of these two viruses were more closely related to chicken-origin ARVs (nt, 76.6–77.5%; aa, 89.3–90.0%) than to classical WRVs (nt, 63.8–68.0%; aa, 76.3–76.7%). Overall, MDRV-SH12 and MDRV-DH13 shared higher sequence identities with WRVs than with chicken-origin ARVs in the M-class segments, except the M2 segment, which showed higher identity with chicken-origin ARVs than with classical WRVs.

The S-class segments showed more divergence than the L- and M-class segments. As shown in Table 2, all S-class segments of MDRV-SH12 and MDRV-DH13 were more closely related to WRVs than to chicken-origin ARVs except the S3 segments, encoding σB proteins, which were more closely related to chicken-origin ARVs than to WRVs. The S2 and S4 segments, which encode the σA and σNS proteins, respectively, shared similar identities with novel WRVs (nt, 87–99%; aa, 96–100%) and classical WRVs (nt, 85–95%; aa, 95–99%), and lower identities with chicken-origin ARVs (nt, 76–78%; aa, 89–92%). For the S1 segment, MDRV-SH12 and MDRV-DH13 showed the highest identities to the novel WRVs HN5d, J18, and ZJ00M, and higher identities to the chicken-origin ARVs D1546 and D2044, which were isolated in France, than with other novel WRVs (data not shown).

### Phylogenetic analyses

To examine the phylogenetic relationships of the two viruses isolated in this study with other duck-, goose-, and chicken-origin ARVs, phylogenetic trees were constructed based on the nucleotide sequences of all 10 genome segments using the Maximum Likelihood method with bootstrapping, and MRVs were included as an outgroup.

**Fig. 1:**
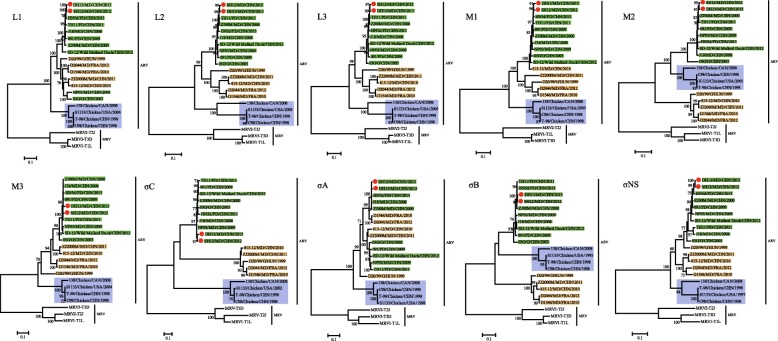
Phylogenetic trees constructed based on the nucleotide sequences of the L, M, and S genome segments of different reoviruses. Phylogenetic calculations were performed by using the maximum-likelihood method applying the best-fit models calculated for each gene. Classical and novel waterfowl-origin reoviruses and chicken-origin reoviruses are indicated as orange, green, and light blue rectangles, respectively, in the phylogenetic trees. The red solid circles indicate the MDRV-SH12 and MDRV-DH13 isolates described in this study. The scale bar is proportional to the genetic distance

As shown in Fig. 1, the phylogenetic trees of individual genes showed divergence between WRVs and chicken-origin ARVs and a close relationship between MDRV-SH12 and MDRV-DH13. These two strains always appeared on the same monophyletic branch, forming common clusters with several Chinese WRVs. For 7 of the 10 genome segments (except M2, S1, and S3), all chicken and waterfowl-origin isolates, including MDRV-SH12 and MDRV-DH13, formed two separate host-associated groups. The phylogenetic tree of the M2 segment showed a different host-independent topological pattern; the two viruses isolated in this study along with the other novel WRVs (ZJ00M, TH11, 091, NP03, HN5d, SD-12, J18, and 03G) were distinct from the classical WRVs (D20/99, 815–12, ZJ2000M, D1546, and D2044) but clustered together with some chicken-origin ARVs (138, C98, S1133, and T-98). Interestingly, in the phylogenetic trees based on the S1 and S3 segments, MDRV-SH12, MDRV-DH13, and other novel WRVs formed a monophyletic group, which was separated not only from the chicken-origin ARVs but also the classical WRVs. In the phylogenetic analysis of segments encoding the λB, λC, μB, σC, and σB genes, MDRV-SH12 and MDRV-DH13 and other novel WRVs were clustered separately from the classical WRVs; however, this grouping pattern was not observed in the phylogenetic trees of the segments encoding λA, μA, μNS, σA, and σNS.

Overall the phylogenetic analyses of the WRVs revealed that the segments encoding μB, σB, and σC exhibited remarkable divergence compared to other segments, as indicated in the pairwise sequence comparisons. For all three segments encoding outer capsid proteins, MDRV-SH12 and MDRV-DH13 were more closely related to previously report novel WRVs, and they formed a monophyletic branch.

### Recombination analyses

**Fig. 2:**
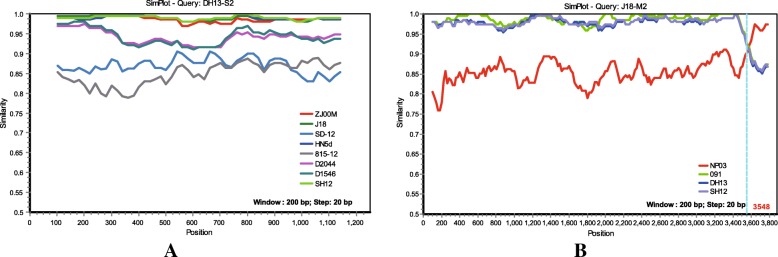
Recombination analyses of all the segment sequences of MDRV-SH12 and MDRV-DH13 and other ARVs were screened using RDP4 and visualized using SimPlot 3.5.1. **a** Recombination analyses of the S2 segments of SH12, DH13, ZJ00M, J18, HN5d, D1546, and D2044. **b** Recombination analyses of the M2 segment of J18

The sequences of all 10 segments of N-MDRV-SH12 and N-MDRV-DH13 and other ARVs were evaluated for the presence of recombination using RDP4 and SimPlot. Recombinant events were detected in the S2 segments of N-MDRV-SH12 and N-MDRV-DH13 and some other WRVs (ZJ00M, J18, HN5d, D1546, and D2044) by RDP4, and the sequences of 815–12 and SD-12, which represent the classical and novel WRVs, respectively, were the parental sequences. However, these recombination events were not significant statistical supported by the RDP and similarity plot analyses (Fig. 2a). Recombination events were also detected in the L1, M1, M2, M3, and S4 segment of some WRVs, including MDRV-SH12 and MDRV-DH13; however, these recombination events also did not receive significant statistical support in the RDP and similarity plot analyses, except the segment L1 of the J18 strain (Fig. 2b). In contrast, multiple methods statistically supported a recombination event in J18, and the recombination breakpoint at position 3548 of the sequence alignment was identified by similarity plot analysis. In the recombination evaluation of the L2, L3, S1, and S3 segments, no recombination event was detected by RDP or similarity plot analysis.

## Discussion

In this study, the complete nucleotide sequences of two novel MDRV isolates, MDRV-SH12 and MDRV-DH13, associated with typical “yellow and white necrotic lesions, with ecchymosis hemorrhages throughout” from Muscovy ducklings in Guangdong province, China were determined, which enabled us to compare these viruses, at the nucleotide and amino acid levels, with other classical and novel WRVs and chicken-origin ARVs. Genomic analysis showed that the lengths of all L-, M-, and S-class segments (except segment S1) of MDRV-SH12 and MDRV-DH13 were almost the same length as other homologous segments of WRVs and chicken-origin ARVs. ORF prediction and homology searches indicated that 9 of the 10 genome segments were monocistronic; the S1 segments of MDRV-SH12 and MDRV-DH13 were tricistronic, and they encoded three partially overlapping ORFs. This finding is similar to what was found for other ARVs, but was distinct from that found for classical WRVs [22, 25, 26].

Pairwise sequence comparisons indicated that MDRV-SH12 and MDRV-DH13 were most similar to each other and that these two viruses were more closely related to novel WRVs based on the nucleotide and amino acid sequence identities of all genome segments. The nucleotide and amino acid sequence identities between these two viruses and the novel and classical WRVs were higher than the identities between these two viruses and chicken-origin ARVs, except for the M2 and S3 segments (Table 2). The genetic divergence of these two viruses from other WRVs and chicken-origin ARVs was confirmed by the phylogenetic analyses of 8 of out 10 segments (except the M2 and S3 segments). These data suggest that WRVs and chicken-origin ARVs may have evolved in a host-dependent manner [22]. The distinct topologies of the phylogenetic analyses of the M2 and S3 segments indicate that a possible re-assortment event may have occurred in segments M2 and S3 between WRVs and chicken-origin ARVs.

Nucleotide sequence comparison of the 10 genome segments showed that the sequence dissimilarities in segments M2, S1, and S3, which encode the structural proteins μB, σC, and σB, respectively, were significantly higher than those of other genes. This finding is reasonable, since μB, σC, and σB are structural proteins that comprise the virion outer capsid and thus are likely under greater selective pressure by the host immune system than other proteins [6, 27]. In particular, the S1 gene showed the highest divergence, which suggests that the S1 gene could serve as a genetic marker for the differentiation and classification of ARVs.

Phylogenetic analysis of individual segments revealed various clustering patterns with reference strains, which in most cases, were supported by high bootstrap values. The host-specific evolution of chicken-origin ARVs and WRVs is obvious in the phylogenetic analyses of most segments, with the exception of the segments encoding μB, σC, and σB, as all novel WRVs clustered together with the classical WRVs and were separated from the chicken-origin ARVs. The segments encoding σC and σB in all novel WRVs formed a monophyletic group separated from both chicken-origin ARVs and classical WRVs, and the μB-encoding M2 segments of all novel WRVs were clustered together with some chicken-origin ARVs but were distinct from classical WRVs, suggesting a possible re-assortment event may have occurred between WRVs and chicken-origin ARVs. Analysis of the λB, λC, μB, σC, and σB genes showed that the novel and classical WRVs were clustered separately, and this grouping pattern was not observed in the phylogenetic trees of λA, μA, μNS, σA, and σNS, suggesting additional re-assortment events between classical and novel WRVs. The results of the recombination analyses were consistent with the phylogenetic analyses, and demonstrated that possible re-assortment events between WRVs and chicken-origin ARVs, and between classical and novel WRVs, may have occurred during the evolution of the ARVs, although some re-assortment events did not receive obvious recombination signal in the RDP analysis and similarity plot analysis. Therefore, the possibility of these events needs to be further evaluated in future studies.

Interestingly, WRVs isolated in different countries did not form distinct lineages. For example, in the phylogenetic analysis of the segment encoding σNS, a classical goose-origin reovirus strain D20/99, isolated in Hungary was clustered with the novel Chinese WRVs (nt identity, 91.1–92.4%; aa identity, 96.5–97.8%). Similar patterns were observed in the phylogenetic analysis of the segment encoding σA. Two classical French Muscovy duck-origin reovirus strains, D1546 and D2044, clustered with some novel Chinese WRVs, including MDRV-SH12 and MDRV-DH13.

## Conclusion

In conclusion, we isolated two viruses from two deceased Muscovy ducklings, and obtained their complete genome sequences. Sequence comparisons and phylogenetic analyses of the 10 genome segments clearly demonstrated that MDRV-SH12 and MDRV-DH13 are novel MDRVs. Although possible re-assortment events were suggested by the phylogenetic analyses, more studies are needed to confirm this. These findings indicate that possible re-assortment between classical and novel ARVs, and between WRVs and chicken-origin ARVs may have occurred in the past. The availability of additional sequences of WRVs from different countries will improve our understanding of the evolutionary relationships among WRVs, and may aid in the development of more effective vaccines against various WRVs.

## Methods

### Clinical samples and virus isolation

Liver samples of two deceased Muscovy ducklings, which were collected by veterinarian of Foshan University from two livestock farms exhibited typical lesions of “White spot disease in Muscovy ducklings” in two different geographical locations of Guangdong province of China, in June 2012 and September 2013, respectively. At necropsy, multiple large yellow necrotic foci were observed both in liver and spleen.

Virus isolation was conducted using embryonated duck eggs, and methods and procedures referred to Yun’s report [28]. Briefly, the liver samples were homogenized in sterile PBS (pH 7.2) containing antibiotics (10,000 units/ml penicillin and 10,000 mg/ml streptomycin) to obtain a 20% suspension (w/v). After centrifuged at 10,000×g for 10 min, the supernatants were filtered through 0.2 μm Supor Membrane Acrodisc Syringe Filter (PALL, Ann Arbor, USA), and inoculated on the chorioallantoic membrane of 10-day-old duck embryos (0.2 mL/embryo). Embryos were incubated at 37 °C and candled twice daily for 5 days. The allantoic fluids, allantoic membranes and embryos of dead duck embryos were harvested, homogenized, and centrifuged. The supernatants were diluted at 1:10 for further passage in the embryos. The supernatant fluid was stored at − 70 °C for further use.

### RNA extraction, genome segments amplification and sequencing

The total RNAs were extracted from the tissues with TRIzol Reagent (Invitrogen, Carlsbad, CA), according to the manufacturer’s instructions and used as a template for reverse-transcription polymerase chain (RT-PCR) with PrimeScript™ One Step RT-PCR Kit (Takara, Dalian, China) following the manufacturer’s instructions. To determine the gene sequences encoding λA, λB, λC, μA, μB, and μNS, P10, P18, σA, σB, σC and σNS, the L/S/M-class genome segments were amplified used the primers listed in Additional file 1: Table S1, consisting of a denaturation step at 94 °C for 5 min followed by 30~35 cycles at 94 °C for 1 min, annealing at a variable temperature (65 °C to 50 °C) for 30 s, extension at 72 °C for 1 min and a final elongation step at 72 °C for 10 min. The RT-PCR products were purified and cloned into pMD18-T vector (TaKaRa Biotechnology Company, Dalian, China) for sequencing with universal M13 forward and reverse primers by Sangon Biotech in Guangzhou.

### Sequence comparisons and phylogenetic analyses

The DNASTAR Lasergene 12 Core Suite was used for Sanger sequencing assembly and nucleotide sequence translation. ORFs were predicted by using ORFfinder (http://www.ncbi.nlm.nih.gov/gorf/gorf.html). Sequence similarity was evaluated by using the BLAST in GenBank (http://blast.ncbi.nlm.nih.gov/Blast.cgi). Sequences were aligned using the ClustalW 1.83 program (http://align.genome.jp/) in MEGA 5.2.

The best-fit evolutionary models for the sequence alignments were determined by using jModel Test version [29] and the General Time Reversible (GTR) nucleotide substitution model with a gamma (Γ)-distribution model of among-site rate variation, and the proportion of invariable sites (i.e., GTR + Γ + I) was found. Phylogenetic trees based on the nucleotide sequences of all 10 genome segments were then constructed by the Maximum Likelihood (ML) method using PhyML v3.0 [30], with bootstrap support values calculated from 1000 replicates. The nucleotide sequence data reported in this study have been deposited in the GenBank database, and the accession numbers of the sequences are listed in Additional file 2: Table S2.

### Recombination detection

The recombination detection was conducted as previously described [31]. Briefly, all segment sequences generated in this study were screened for recombination by using the RDP, GENECONV, BootScan methods in Recombination Detection Program, version 4 (RDP4) [32]. The significant evidence (*P* < 0.05) of recombination detected by at least two methods and confirmed by phylogenetic analysis was taken to represent strong evidence for recombination. Additionally, the parent strains of the recombination determined above were visualized by using the similarity plot analysis in SimPlot Version 3.5.1 [33], with a window size of 200 bp and a step size of 20 bp.

## Additional files

Additional file 1: **Table S1.** Oligonucleotide primers used to amplify and sequence the L/S/M-class genes of novel pathogen Muscovy duck reovirus (N-MDRV) SH12 and DH13. (DOCX 17 kb)

Additional file 2: **Table S2.** General information of sequences used in this study. (DOCX 25 kb)

## Abbreviations

ARV: Avian reovirus
BRV: Baboon reovirus
dsRNA: double-stranded RNA
MDRV: Muscovy duck reovirus
MRV: mammalian reovirus
NBV: Nelson Bay reovirus
NCRs: non-coding regions
NDRV: Novel duck reovirus
N-MDRV: novel Muscovy duck reovirus
ORF: open reading frame
RRV: Reptilian reovirus
RT-PCR: reverse-transcription polymerase chain
UTRs: un-translated regions
WRV: Waterfowl-origin reovirus
