# Supplementary material for: Molecular characterization of two novel reoviruses isolated from Muscovy ducklings in Guangdong, China
Source: BMC Vet Res. 2019 May 10;15:143. doi: 10.1186/s12917-019-1877-x (PMC6511161; doi:10.1186/s12917-019-1877-x)
Supplement: Supplementary file 1 — Table S1. Oligonucleotide primers used to amplify and sequence the L/S/M-class genes of novel pathogen Muscovy duck reovirus (N-MDRV) SH12 and DH13. (DOCX 17 kb) [file 12917_2019_1877_MOESM1_ESM.docx]

| Primers | Oligonucleotide sequence（5’➞3’） | Length of segments（bp） |
| --- | --- | --- |
| S1-F | GCTTTTTTCTTCTCTGCCCAT | 1568 |
| S1-R | GATGAATAGCTCTTCTCATCGTGC |  |
| S2-F | GCTTTTTCTCCCACGATGGC | 1324 |
| S2-R | GATGAATACACCCACGCGCTAC |  |
| S3-F | GCTTTTTGAGTCCTCAGCGTG | 1202 |
| S3-R | GATGAATAGGCGAGTCCCGC |  |
| S4-F | GCTTTTTGAGTCCTTGTGCA | 1191 |
| S4-R | GATGAATAAGAGTCCAAGTCGC |  |
| M1-F | GCTTTTCTCGACATGGCCTATCTAGC | 2284 |
| M1-R | GATGAATATCTCAAGACGGCTAACCCAGG |  |
| M2-F | GCTTTTTGAGTGCTAACCT | 2158 |
| M2-R | GATGAGTAACGTGCTAACC |  |
| M3-F | GCTTTTTGAGTCCTAGCGTGG | 1996 |
| M3-R | GATGAGTAACCGAGTCCGCCGTGG |  |
| L1a-F | GCTTTTTCTCCGAACGCCGA | 2041 |
| L1a-R | TAGGGTCATCCATAGGCAAATTCTC |  |
| L1b-F | CCTATGGATGACCCTAACTT | 1934 |
| L1b-R | GATGAATAACCTCCAACGA |  |
| L2a-F | GCTTTTTCCTCACCATGCAT | 1958 |
| L2a-R | TGACACATAACCTGGAAACC |  |
| L2b-F | GTCCTCAATGCCTATTTCCG | 1913 |
| L2b-R | GATGAGTAATTCCTCGAGCCA |  |
| L3a-F | GCTTTTACACCCATGGCTCA | 2118 |
| L3a-R | AGTGGGTCGTCCAGCGTAA |  |
| L3b-F | CTTTCAATCCCTCCGCTG | 1921 |
| L3b-R | GATGAGTAACACCCTTCTACTGGAG |  |

Table S1 Oligonucleotide primers used to amplify and sequence the L/S/M-class genes of novel pathogen Muscovy duck reovirus (N-MDRV) SH12 and DH13.
