# Supplementary material for: Molecular characterization of two novel reoviruses isolated from Muscovy ducklings in Guangdong, China
Source: BMC Vet Res. 2019 May 10;15:143. doi: 10.1186/s12917-019-1877-x (PMC6511161; doi:10.1186/s12917-019-1877-x)
Supplement: Supplementary file 2 — Table S2. General information of sequences used in this study. (DOCX 25 kb) [file 12917_2019_1877_MOESM2_ESM.docx]

| Strain | p10/p17/σC（S1） | σA（S2） | σB（S3） | σNS（S4） | μA（M1） | μB（M2） | μNS（M3） | λA（L1） | λB（L2） | λC（L3） | Origin | Host |
| --- | --- | --- | --- | --- | --- | --- | --- | --- | --- | --- | --- | --- |
| ZJ00M | KF154116 | KF154117 | KF154118 | KF154119 | KF154113 | KF154114 | KF154115 | KF154110 | KF154111 | KF154112 | China | Muscovy duck |
| TH11 | KC493571 | JQ664689 | JX826588 | JX826589 | JX440513 | JX440514 | JX440512 | KC493572 | KC493573 | KC493574 | China | Pekin duck |
| 091 | JX478256 | JX478257 | JX478258 | JX478259 | JX478253 | JX478254 | JX478255 | JX478250 | JX478251 | JX478252 | China | Pekin duck |
| NP03 | KC312699 | JF320803 | GQ888710 | GU338025 | JF320802 | JF320801 | JF320800 | KC312700 | KC312701 | KC312702 | China | Muscovy duck |
| HN5d | KT861593 | KT861594 | KT861595 | KT861596 | KT861590 | KT861591 | KT861592 | KT861587 | KT861588 | KT861589 | China | Pekin duck |
| SD-12 | KJ879930 | KJ879931 | KJ879932 | KJ879933 | KJ879927 | KJ879928 | KJ879929 | KJ879924 | KJ879925 | KJ879926 | China | Wild Mallard Duck |
| J18 | JX478266 | JX478267 | JX478268 | JX478269 | JX478263 | JX478264 | JX478265 | JX478260 | JX478261 | JX478262 | China | Muscovy duck |
| 03G | JX145334 | JX145336 | JX145336 | JX145337 | JX145331 | JX145332 | JX145333 | JX145333 | JX145329 | JX145330 | China | Goose |
| D20/99 | KF809668 | KF809669 | KF809670 | KF809671 | KF809665 | KF809666 | KF809667 | KF809662 | KF809663 | KF809664 | Hungary | Goose |
| 815-12 | KC508656 （S4） | KC508653  （S1） | KC508654（S2） | KC50865（S3） | KC508650 | KC508651 | KC508652 | KC508647 | KC508648 | KC508649 | China | Muscovy duck |
| ZJ200M | KF306091（S4） | KF306088（S1） | KF306089（S2） | KF306091（S3） | KF306085 | KF306086 | KF306087 | KF306082 | KF306083 | KF306084 | China | Muscovy duck |
| D1546 | KJ871023 | KJ871024 | KJ871025 | KJ871026 | KJ871020 | KJ871021 | KJ871022 | KJ871017 | KJ871019 | KJ871018 | France | Muscovy duck |
| D2044 | KJ871013 | KJ871014 | KJ871015 | KJ871016 | KJ871010 | KJ871011 | KJ871012 | KJ871007 | KJ871009 | KJ871008 | France | Muscovy duck |
| 138 | AF218359 | AF059717 | AF059721 | AF059725 | AY557188 | AY750052 | AY557190 | EU707933 | EU707935 | EU707937 | Canada | Chicken |
| C98 | EF057397 | JN641886 | EF030496 | JN641885 | EU616740 | EU616741 | EU616744 | EU616735 | JN641888 | EU616737 | China | Chicken |
| S1133 | KF741762 | KF741763 | KF741764 | KF741765 | KF741759 | KF741760 | KF741761 | KF741756 | KF741757 | KF741758 | USA | Chicken |
| T-98 | EF057398 | JN641887 | EF030499 | JN641884 | EU616736 | EU616742 | EU616743 | EU616739 | JN641889 | EU616738 | China | Chicken |

Table S2 General information of sequences used in this study.
